# Clinical characteristics of non-small cell lung cancer harboring mutations in exon 20 of *EGFR* or *HER2*

**DOI:** 10.18632/oncotarget.24958

**Published:** 2018-04-20

**Authors:** Masayuki Takeda, Kazuko Sakai, Hidetoshi Hayashi, Kaoru Tanaka, Junko Tanizaki, Takayuki Takahama, Koji Haratani, Kazuto Nishio, Kazuhiko Nakagawa

**Affiliations:** ^1^ Department of Medical Oncology, Kindai University Faculty of Medicine, Osaka-Sayama, Osaka 589-8511, Japan; ^2^ Department of Genome Biology, Kindai University Faculty of Medicine, Osaka-Sayama, Osaka 589-8511, Japan

**Keywords:** epidermal growth factor receptor gene (EGFR), human epidermal growth factor receptor 2 gene (HER2), resistance, lung cancer, nivolumab

## Abstract

Unlike common epidermal growth factor receptor gene (*EGFR*) mutations that confer sensitivity to tyrosine kinase inhibitors (TKIs) in non-small cell lung cancer (NSCLC), mutations in exon 20 of either *EGFR* or the human EGFR2 gene (*HER2*) are associated with insensitivity to EGFR-TKIs, with treatment options for patients with such mutations being limited. Clinical characteristics, outcome of EGFR-TKI or nivolumab treatment, and the presence of coexisting mutations were reviewed for NSCLC patients with exon-20 mutations of *EGFR* or *HER2* as detected by routine application of an amplicon-based next-generation sequencing panel. Between July 2013 and June 2017, 206 patients with pathologically confirmed lung cancer were screened for genetic alterations including *HER2* and *EGFR* mutations. Ten patients harbored *HER2* exon-20 insertions (one of whom also carried an exon-19 deletion of *EGFR*), and 12 patients harbored EGFR exon-20 mutations. Five of the 13 patients with *EGFR* mutations were treated with EGFR-TKIs, two of whom manifested a partial response, two stable disease, and one progressive disease. Among the seven patients treated with nivolumab, one patient manifested a partial response, three stable disease, and three progressive disease, with most (86%) of these patients discontinuing treatment as a result of disease progression within 4 months. The H1047R mutation of *PIK3CA* detected in one patient was the only actionable mutation coexisting with the exon-20 mutations of *EGFR* or *HER2*. Potentially actionable mutations thus rarely coexist with exon-20 mutations of *EGFR* or *HER2*, and EGFR-TKIs and nivolumab show limited efficacy in patients with such exon-20 mutations.

## INTRODUCTION

Lung cancer is the most common cause of cancer-related death worldwide, with non-small cell lung cancer (NSCLC) accounting for ~75% of all lung cancer cases [1]. Recent insight into the molecular basis of lung cancer has led to changes in disease treatment. The identification of driver mutations, such as those affecting the epidermal growth factor receptor (EGFR) [2–4] and anaplastic lymphoma kinase (ALK) [5, 6] genes, has already been successfully translated into clinical practice. Most patients with NSCLC harboring *EGFR* mutations show an initial marked response to EGFR tyrosine kinase inhibitors (TKIs) [7]. Exon-19 deletions (Ex19del) and the L858R point mutation in exon 21 of *EGFR* confer high sensitivity to EGFR-TKIs and account for ~85% of all *EGFR* mutations [8]. The remaining *EGFR* mutations include point mutations in exon 18 (G719X, ~3% of *EGFR* mutations) and exon 21 (L861Q, ~2% of *EGFR* mutations) that confer moderate sensitivity to EGFR-TKIs. On the other hand, exon-20 insertions account for ~5% of all *EGFR* mutations and define a distinct subset of lung adenocarcinoma characterized by a poor response to the first-generation EGFR-TKIs gefitinib and erlotinib [9–11].

Even patients with TKI-sensitizing *EGFR* mutations can manifest *de novo* resistance or only a short-term response to EGFR-TKIs. One mechanism of such *de novo* resistance is mediated by a T790M mutation in exon 20 of *EGFR* that impedes drug binding to the ATP pocket of the receptor protein [12, 13]. The S768I mutation in exon 20 of *EGFR* has similarly been associated with a poor response to EGFR-TKI treatment in a case series [14]. Mutations of the human epidermal growth factor receptor 2 gene (*HER2*, also known as *ERBB2*) have also been identified as oncogenic drivers in NSCLC with a frequency of 2% to 3%, with most such mutations consisting of a 12-bp in-frame insertion (encoding YVMA) in exon 20 [15–17]. Preclinical and clinical studies have shown that such mutations in the kinase domain result in constitutive phosphorylation and activation of HER2 [18]. Similar to patients with *EGFR* exon-20 mutations, treatment with the EGFR- and HER2-selective TKIs afatinib or dacomitinib has met with limited success in patients harboring exon-20 mutations of *HER2* [17, 19].

Immune-checkpoint inhibitors such as antibodies to programmed cell death–1 (PD-1) have emerged as promising therapeutic agents for NSCLC. Phase III trials of the PD-1 inhibitors nivolumab and pembrolizumab in previously treated patients with NSCLC have thus indicated that immunotherapy strategies will provide new therapeutic options for advanced NSCLC [20–22]. Biomarker studies revealed a significant correlation between the pretreatment expression level of the PD-1 ligand PD-L1 in tumor cells (as determined by immunohistochemical analysis) and the likelihood of a response to PD-1 inhibitors, whereas *EGFR* mutations appear to be a negative predictive factor for PD-1 inhibitor efficacy. Given that most *EGFR* mutations are either exon-19 deletions or L858R in exon 21, however, it has been unclear whether such treatment is also without benefit in patients with uncommon *EGFR* mutations.

Given the limited treatment options for NSCLC patients harboring mutations in exon 20 of either *EGFR* or *HER2*, it is important that the clinical features and treatment response of such patients be further explored. We have therefore now evaluated the clinical background of patients with NSCLC positive for *EGFR* or *HER2* exon-20 mutations as well as their response to treatment including that with the immune-checkpoint inhibitor nivolumab. We also examined the patients for coexisting mutations that offer the potential for targeted treatment.

## RESULTS

### Patient characteristics

Between July 2013 and June 2017, 206 patients with pathologically confirmed lung cancer were screened with a next-generation sequencing (NGS) panel including *HER2* and *EGFR* genes at Kindai University Hospital (Supplementary Figure 1). Nine patients (4%) were found to harbor *HER2* exon-20 mutations, 12 patients (6%) to harbor *EGFR* exon-20 mutations, and one patient (0.5%) to harbor a *HER2* exon-20 mutation as well as an *EGFR* Ex19del mutation. The demographics of these 22 patients included in the present study are shown in Table 1. Thirteen (59%) of the patients were female, and 15 (68%) were never- or light smokers, with the median age of all patients being 70 years (range, 44 to 81). Most (91%) patients had a good Eastern Cooperative Oncology Group performance status (0 or 1) at the onset of initial chemotherapy. Twenty-one patients (95%) had adenocarcinoma, and 18 (82%) had disease of stage IV. With regard to the type of *EGFR* exon-20 mutations (*n* = 12), eight patients (36%) had exon-20 insertions, three (14%) had a known exon-20 resistance mutation (T790M or S768I) together with a TKI-sensitizing mutation (Ex19del, G719C, or L858R), and one (5%) had a G779F mutation, whose effect on TKI sensitivity is unknown. All 10 patients with *HER2* exon-20 mutations harbored in-frame insertions (A775_G776 ins YVMA in nine patients, and A775_G776 ins V in one).

**Table 1:** Characteristics of NSCLC patients harboring exon-20 mutations of *EGFR* or *HER2* (*n* = 22)

Characteristic	Subset	No. of patients (%)
[Median (range) age in years		70 (44–81)]
Sex	Male	9 (41)
	Female	13 (59)
Smoking status	Never	14 (64)
	Light smoker^a^	1 (5)
	Smoker^a^	7 (32)
ECOG PS	0	4 (18)
	1	16 (73)
	2	2 (9)
Tumor histology	Adenocarcinoma	21 (95)
	NSCLC, favoring adenocarcinoma	1 (5)
Disease stage	IIIA	1 (5)
	IV	18 (82)
	Postoperative recurrence	3 (14)
*EGFR* exon-20 mutation	M766_A767 ins ASV	2 (9)
(*n* = 12)	A767_S768 ins SVD	2 (9)
	A767_S768 ins SVG	1 (5)
	S768I (+ G719C)	1 (5)
	S768I (+ L858R)	1 (5)
	V769_D770 ins ASV	1 (5)
	D770_N771 ins GL	1 (5)
	D770_N771 ins NPG	1 (5)
	G779F	1 (5)
	T790M (+ E746_T751 delins A)	1 (5)
*HER2* exon-20 mutation	A775_G776 ins V	1 (5)
(*n* = 9)	A775_G776 ins YVMA	8 (36)
*EGFR/HER2* double	*EGFR* E746_A750 del +	1 (5)
mutation (*n* = 1)	*HER2* A775_G776 ins YVMA	

^a^Light smoker, ≤10 pack-years; smoker, >10 pack-years. ECOG PS, Eastern Cooperative Oncology Group performance status.

**Figure 1:**
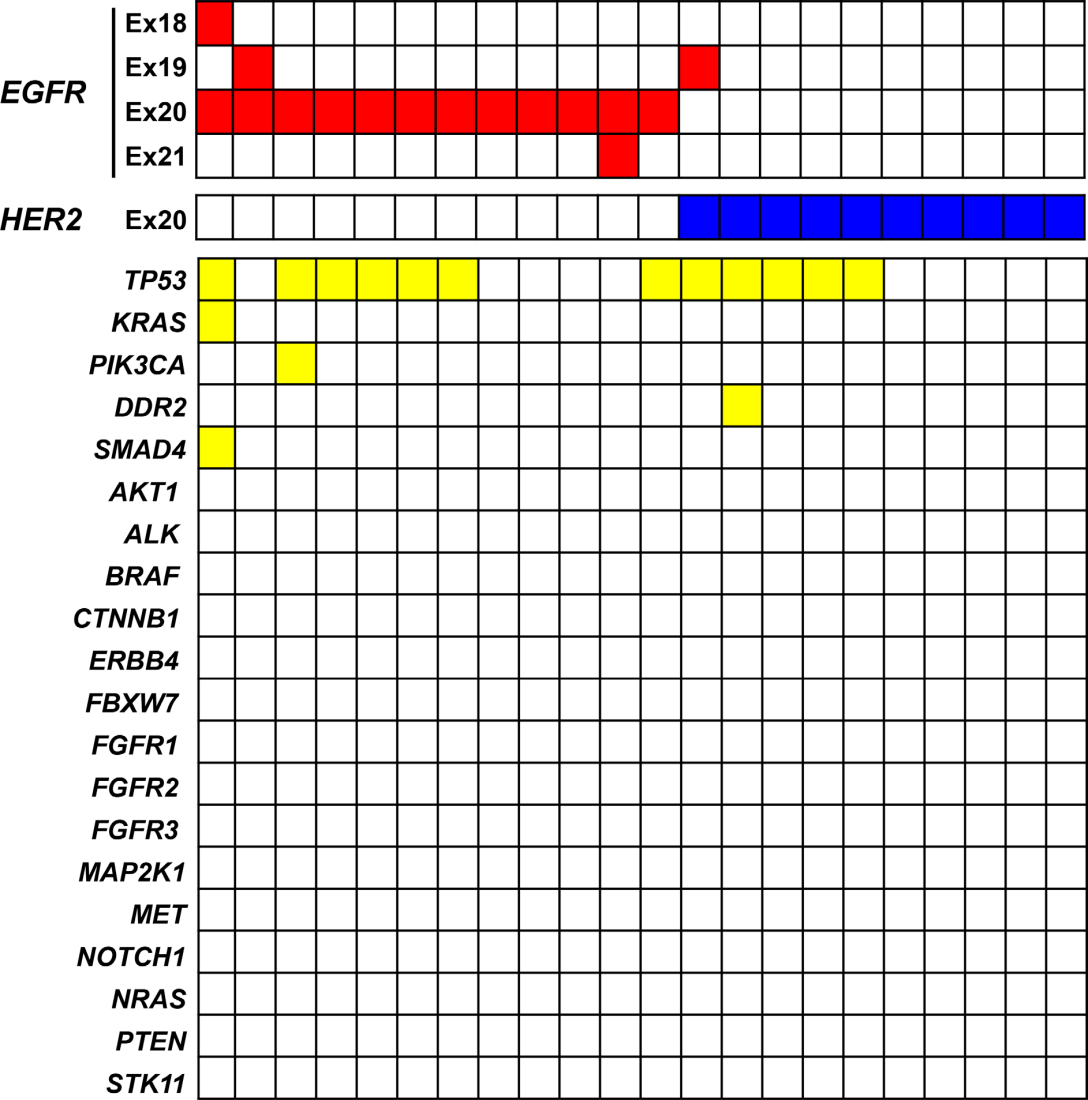
Somatic gene mutations detected with an NGS panel covering 22 genes in lung cancer specimens positive for exon-20 mutations of *EGFR* or *HER2* Each column corresponds to one of the 22 patients.

A total of 16 nonsynonymous mutations in genes other than *EGFR* and *HER2* was detected in the 22 patients, for a mean of 0.7 such mutations per tumor (Figure 1). The most common such genetic alterations were *TP53* mutations, which were detected in 12 patients (55%), whereas *SMAD4*, *PIK3CA*, *DDR2*, and *KRAS* mutations were each present in one patient (5%). Among these various genetic alterations, only one (H1047R of *PIK3CA*) was potentially treatable with therapeutic agents.

### Outcome of EGFR-TKI treatment

Median overall survival (OS) for all patients (*n* = 22) harboring *EGFR* or *HER2* exon-20 mutations was 23.5 months, with 13 deaths and a median follow-up time of 18.2 months for survivors at last censoring. Most patients (*n* = 16, 73%) received platinum-based chemotherapy as a first-line drug treatment: pemetrexed plus platinum, with or without bevacizumab, in 13 patients, paclitaxel plus platinum in two patients, and S-1 plus platinum plus bevacizumab in one patient (Supplementary Figure 1). Of the remaining patients (*n* = 6), two individuals received an EGFR-TKI, two pemetrexed, one docetaxel, and one best supportive care.

**Figure 2:**
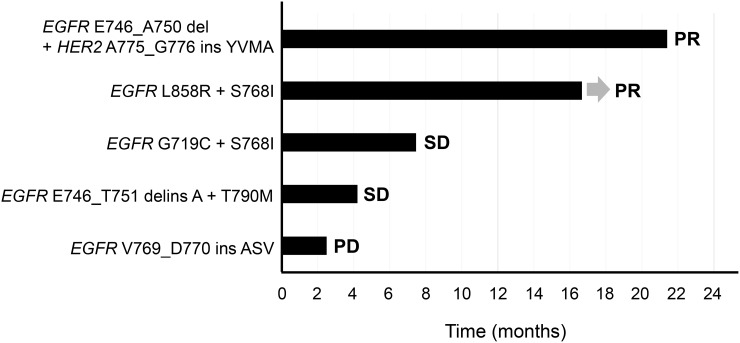
Swimmer plot for duration of disease stability or response to EGFR-TKI treatment in patients with exon-20 mutations of *EGFR* or *HER2* Bar length indicates the duration of EGFR-TKI treatment for each patient, with the best response observed before treatment failure indicated on the right. The origin corresponds to treatment start date, and the arrow indicates an ongoing response at the time of data censoring.

Among the 13 patients positive for *EGFR* mutations, five individuals received an EGFR-TKI, including three treated with a first-generation agent (gefitinib or erlotinib) and two with a second-generation agent (afatinib). Two of these five patients received the EGFR-TKI as first-line chemotherapy, whereas the remaining three received at least one cytotoxic chemotherapy regimen, including platinum-based combinations, before EGFR-TKI treatment. Two of the five patients manifested a partial response (PR) to EGFR-TKI treatment, two stable disease (SD), and one progressive disease (PD), with the progression-free survival (PFS) of all five individuals during EGFR-TKI therapy being shown in Figure 2. The two patients who achieved a response with long-term remission (>1 year) harbored common TKI-sensitizing *EGFR* mutations (*EGFR* E746_A750 del + *HER2* A775_G776 ins YVMA in one, and *EGFR* L858R + S768I in the other).

### Outcome of nivolumab treatment

**Figure 3:**
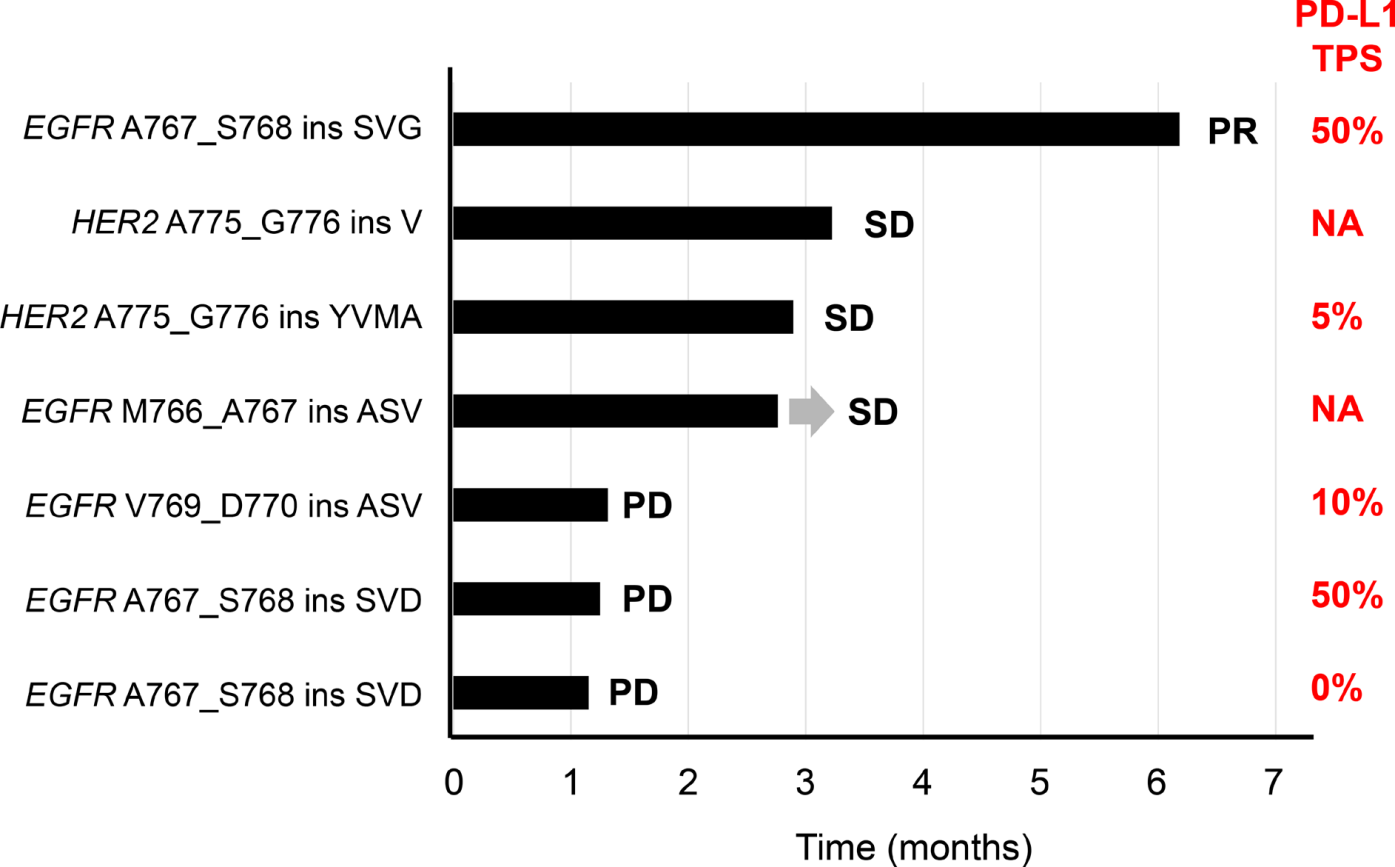
Swimmer plot for duration of disease stability or response to nivolumab treatment in patients with exon-20 mutations of *EGFR* or *HER2* Bar length indicates the duration of nivolumab treatment for each patient, with the best response observed before treatment failure indicated on the right. The origin corresponds to treatment start date, and the arrow indicates an ongoing response at the time of data censoring. The TPS for PD-L1 expression in tumor specimens is indicated at the far right. NA, not assessed.

Given the limited efficacy of EGFR-TKIs for the distinct subset of NSCLC patients with *EGFR* exon-20 mutations as well as the poor response to EGFR-TKI treatment apparent for NSCLC patients with *HER2* exon-20 mutations, we examined the efficacy of immune-checkpoint inhibitors in these patients. Five patients with *EGFR* exon-20 insertions and two with *HER2* exon-20 insertions received nivolumab therapy. Five of these seven patients received nivolumab as second-line chemotherapy, whereas the remaining two patients received the drug in the third-line or later setting (Supplementary Figure 1). Among the seven patients treated with nivolumab, one manifested a PR, three SD, and three PD, with the PFS for all these individuals during nivolumab therapy being shown in Figure 3. Most (86%) patients discontinued nivolumab treatment as a result of disease progression within 4 months. The tumors of five patients who received nivolumab therapy were assessed for PD-L1 expression by immunohistochemistry. The tumor proportion score (TPS) for PD-L1 expression [23] was 50% in two patients and 10%, 5%, or 0% in the remaining three (Figure 3).

## DISCUSSION

Most NSCLC patients harboring TKI-sensitizing *EGFR* mutations show an initial marked response to EGFR-TKI treatment. The clinical course of *EGFR* mutation-positive NSCLC during EGFR-TKI treatment shows substantial variability, however [7]. Insertions in exon 20 of *EGFR* have thus been associated with resistance to EGFR-TKIs in NSCLC patients [9–11]. Case-series studies have found the objective response rate for EGFR-TKI (gefitinib or erlotinib) treatment in patients with *EGFR* exon-20 mutations to be ~10% to 20%, with a median PFS of ~2 months [9, 11, 14]. The OS of patients with tumors harboring *EGFR* exon-20 insertions was found to be similar to that for those with tumors wild-type for *EGFR* [11]. Afatinib was also shown to have limited efficacy in patients with *EGFR* exon-20 insertions [24]. In addition, a *de novo* T790M mutation or an S768I mutation in exon 20 of *EGFR* is associated with moderate efficacy of or resistance to EGFR-TKI treatment in patients whose tumors also harbor TKI-sensitizing *EGFR* mutations [12–14]. Consistent with these previous observations, our retrospective case series has shown that a patient with an *EGFR* exon-20 insertion did not respond to EGFR-TKI treatment, although two patients harboring common TKI-sensitizing *EGFR* mutations together with other resistance mutations achieved a long-term response to such treatment.

The prevalence of *HER2* mutations has been found to range from 2% to 3% in NSCLC [15–17]. In the present study, *HER2* mutations were detected in 5% of NSCLC patients and were associated with never-smoker status, female sex, and adenocarcinoma histology, consistent with previous observations. A phase II trial of dacomitinib, an irreversible TKI with activity against both EGFR and HER2, revealed that only 12% of NSCLC patients harboring *HER2* exon-20 mutations experienced a PR, with none of those harboring a YVMA insertion showing a response [17]. Several next-generation HER2-targeted drugs, such as trastuzumab and trastuzumab emtansine (T-DM1), have been developed to overcome such resistance [25, 26]. Patients with tumors positive for *HER2* mutations in the present study were not able to access clinical trials evaluating the efficacy of such HER2-targeted agents for *HER2* mutant tumors in Japan (University Medical Hospital Information Network [UMIN] Clinical Trials Registry identifiers UMIN000012551 and UMIN000017709) because of the close of enrollment.

Immunotherapy offers a novel approach to the treatment of NSCLC patients. Immune-checkpoint inhibitors, including antibodies to PD-1 such as nivolumab and pembrolizumab, have been introduced into clinical practice for the treatment of advanced NSCLC on the basis of the results of large phase III studies showing that they confer increased OS compared with standard second-line docetaxel [20–22]. However, the efficacy of such agents tends to be less pronounced in patients with common *EGFR* mutations, possibly as a result of a low level of mutational heterogeneity [27], with somatic mutation load being thought to be correlated with sensitivity to immune-checkpoint inhibitors. Given the rarity of exon-20 mutations of *EGFR* and *HER2* relative to the major activating mutations of *EGFR*, clinical information regarding the relation between these exon-20 mutations and the response to nivolumab has not been available. Our study has now shown limited success for nivolumab treatment in patients with exon-20 mutations of *EGFR* or *HER2*, similar to the case for those with common *EGFR* mutations such as Ex19del and L858R.

Given the limited therapeutic efficacy of EGFR-TKIs and immune-checkpoint inhibitors in patients with exon-20 mutations of *EGFR* or *HER2*, the identification of actionable mutations should be considered for such individuals in order to provide additional therapy options. Exon-20 mutations of *EGFR* and *HER2* were previously found to be mutually exclusive with other driver alterations such as mutations in *KRAS* and *BRAF* [28, 29]. To identify additional oncogenic mutations, we prospectively applied an amplicon-based NGS panel that covers mutational hotspots related to lung and colon tumorigenesis. Most coexisting mutations were found to be located in *TP53*, with only the H1047R mutation of *PIK3CA* being considered actionable as a result of its association with the therapeutic efficacy of PI3K-AKT-mTOR pathway inhibitors [30]. Other somatic mutations detected in *SMAD4*, *KRAS*, and *DDR2* are thought to be passenger mutations. A limitation of our study is that detection of other coexisting mutations was limited to those covered by the relatively small NGS panel. Given that *EGFR*-mutated lung cancer was previously shown in a study based on NGS to have a lower mutation burden compared with lung cancer wild-type for *EGFR* [27], it may be difficult to identify concurrent actionable driver mutations in patients with *EGFR* or *HER2* mutations. Moreover, the NGS analysis is performed mostly by K.S. (second author of this manuscript). M.T. (first author) is largely responsible for writing an annotation report for each patient and for establishment and maintenance of a database. Therefore, gathering of data by one investigator may produce unbiased results.

Given that the outcome for NSCLC patients with uncommon *EGFR* or *HER2* mutations remains poor, with only a moderate at best benefit conferred by treatment with EGFR-TKIs or immune-checkpoint inhibitors and with the rarity of coexisting actionable mutations, novel therapies that improve outcome in these patients are urgently needed.

## PATIENTS AND METHODS

### Patients

The study population was selected from the cohort of patients enrolled in an ongoing observational study of the clinical application of NGS to therapeutic decision-making for lung cancer at Kindai University Hospital (UMIN000014782), the design and initial results of which have been presented previously [31]. In brief, the study enrolled patients about to undergo either biopsy for the purpose of diagnosis of or curative surgery for lung cancer, or those with already histologically proven lung cancer. No restrictions on tumor histology, disease stage, subsequent or previous treatment, performance status, or other factors were imposed. Lung cancer staging was performed in all patients according to the 7th TNM classification. All patients provided written informed consent to the performance of genomics analysis, and the study was approved by the Institutional Review Board of Kindai University. Genetic alterations were defined as actionable if they were associated with susceptibility to an approved targeted therapy or if they could serve as a basis for direction of patients toward registered clinical trials (either nationally or at our institution). Tumor response was examined by computed tomography and was evaluated according to the Response Evaluation Criteria in Solid Tumors (RECIST) version 1.1. A best response of SD required the criterion to be met for at least 6 weeks after initiation of treatment.

### Molecular testing

Collected formalin-fixed, paraffin-embedded tumor specimens underwent histological review, and only those containing sufficient tumor cells as revealed by hematoxylin-eosin staining were subjected to nucleic acid extraction. DNA was purified with the use of an Allprep DNA FFPE Kit (Qiagen), and 10 ng of the purified material were subjected to multiplex polymerase chain reaction (PCR) amplification and NGS with the use of an Ion AmpliSeq Library Kit 2.0 and Ion AmpliSeq Colon and Lung Cancer Panel (Life Technologies), which targets 504 mutational hotspot regions of 22 cancer-associated genes: *AKT1*, *ALK*, *BRAF*, *CTNNB1*, *DDR2*, *EGFR*, *ERBB2*, *ERBB4*, *FBXW7*, *FGFR1*, *FGFR2*, *FGFR3*, *KRAS*, *MAP2K1*, *MET*, *NOTCH1*, *NRAS*, *PIK3CA*, *PTEN*, *SMAD4*, *STK11*, and *TP53* [31, 32]. DNA sequencing data were accessed through the Torrent Suite v.4.0 program (Life Technologies). Reads were aligned with the hg19 human reference genome, and variants were called with the use of Variant Call Format ver. 4.0. Raw variant calls were filtered with the use of the following annotations: homozygous and heterozygous variants, quality score of <100, and depth of coverage of <19. The tumors of five patients who received nivolumab therapy were assessed for PD-L1 expression by immunohistochemistry with the 22C3 antibody as performed as part of clinical practice and in a commercial clinical laboratory (SRL, Tokyo). Scoring for the PD-L1 IHC 22C3 pharmDx assay in NSCLC is based on the percentage of stained tumor cells, or TPS [23].

### Statistical analysis

The present observational study was undertaken without prior determination of sample size or power. The primary end point of the study was EGFR-TKI or nivolumab efficacy in patients with NSCLC positive for *EGFR* or *HER2* exon-20 mutations. PFS was calculated from the date of initiation of chemotherapy either to the date of disease progression or to the date of last contact. OS was calculated from the date of diagnosis of metastatic cancer or of recurrence after curative surgery to the date of death from any cause or to the date of last contact. The probability of survival as a function of time was estimated with the Kaplan-Meier method as performed with GraphPad Prism software version 5.0.

## SUPPLEMENTARY MATERIALS AND FIGURES


