# Supplementary material for: Clinical characteristics of non-small cell lung cancer harboring mutations in exon 20 of EGFR or HER2
Source: Oncotarget. 2018 Apr 20;9(30):21132–40. doi: 10.18632/oncotarget.24958 (PMC5940408; doi:10.18632/oncotarget.24958)
Supplement: Supplementary file 1 [file oncotarget-09-21132-s001.pdf]

Clinical characteristics of non–small cell lung cancer harboring mutations in exon 20 of *EGFR* or *HER2*

SUPPLEMENTARY MATERIALS

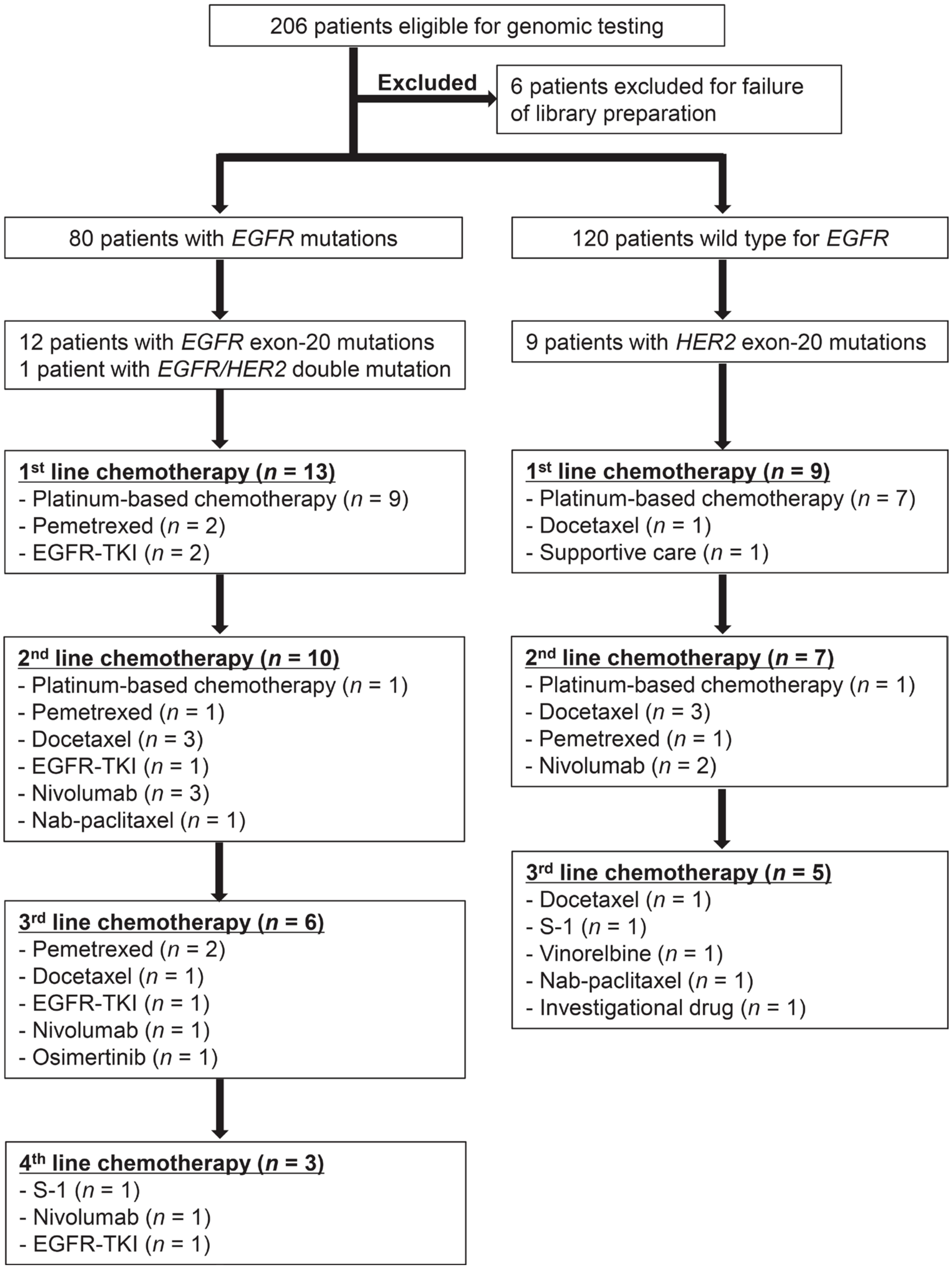

Supplementary Figure 1: Patient flow diagram. EGFR-TKI refers to first- or second-generation drugs.
